# Whole genome sequencing of a single *Bos taurus *animal for single nucleotide polymorphism discovery

**DOI:** 10.1186/gb-2009-10-8-r82

**Published:** 2009-08-06

**Authors:** Sebastian H Eck, Anna Benet-Pagès, Krzysztof Flisikowski, Thomas Meitinger, Ruedi Fries, Tim M Strom

**Affiliations:** 1 Institute of Human Genetics, Helmholtz Zentrum München, German Research Center for Environmental Health, Ingolstädter Landstr., 85764 Neuherberg, Germany; 2 Lehrstuhl für Tierzucht, Technische Universität München, Hochfeldweg, 85354 Freising-Weihenstephan, Germany; 3 Institute of Human Genetics, Klinikum rechts der Isar, Technische Universität München, Trogerstr., 81675 München, Germany

## Abstract

**Background:**

The majority of the 2 million bovine single nucleotide polymorphisms (SNPs) currently available in dbSNP have been identified in a single breed, Hereford cattle, during the bovine genome project. In an attempt to evaluate the variance of a second breed, we have produced a whole genome sequence at low coverage of a single Fleckvieh bull.

**Results:**

We generated 24 gigabases of sequence, mainly using 36-bp paired-end reads, resulting in an average 7.4-fold sequence depth. This coverage was sufficient to identify 2.44 million SNPs, 82% of which were previously unknown, and 115,000 small indels. A comparison with the genotypes of the same animal, generated on a 50 k oligonucleotide chip, revealed a detection rate of 74% and 30% for homozygous and heterozygous SNPs, respectively. The false positive rate, as determined by comparison with genotypes determined for 196 randomly selected SNPs, was approximately 1.1%. We further determined the allele frequencies of the 196 SNPs in 48 Fleckvieh and 48 Braunvieh bulls. 95% of the SNPs were polymorphic with an average minor allele frequency of 24.5% and with 83% of the SNPs having a minor allele frequency larger than 5%.

**Conclusions:**

This work provides the first single cattle genome by next-generation sequencing. The chosen approach - low to medium coverage re-sequencing - added more than 2 million novel SNPs to the currently publicly available SNP resource, providing a valuable resource for the construction of high density oligonucleotide arrays in the context of genome-wide association studies.

## Abstract 2

The next generation sequencing of a single cow genome with low-to-medium coverage has revealed 2.44 million new SNPs.

## Background

The bovine reference genome sequence assembly resulted from the combination of shotgun and bacterial artificial chromosome sequencing of an inbred Hereford cow and her sire using capillary sequencing. Most of the more than 2 million bovine SNPs deposited in dbSNP represent polymorphisms detected in these two Hereford animals [1]. Recently, Van Tassell *et al*. [2] contributed more than 23,000 SNPs to the bovine SNP collection by next-generation sequencing of reduced representation libraries. The study involved 66 cattle representing different lines of a dairy breed (Holstein) and the 7 most common beef breeds (Angus, Red Angus, Charolais, Gelbvieh, Hereford, Limousin and Simmental). These SNPs together with SNPs deposited in dbSNP were used to compile arrays with up to 50,000 SNPs. The arrays have been used to implement a new approach to animal breeding, termed genomic selection [3,4]. Although this approach has been applied successfully to predict breeding values in dairy cattle, the underlying SNP resource is far from complete. SNP selection for the Illumina BovineSNP50 array, for instance, has been optimized to provide high minor allele frequencies (MAFs) for the Holstein breed. The full extent of common SNP variation in Holstein and other breeds is still unexplored. Although the average r^2 ^between adjacent markers of the BovineSNP50 array is greater than 0.2 - the minimal linkage disequilibrium required for genomic prediction to be sufficiently accurate - there is a considerable number of marker pairs with an r^2 ^of zero [3]. Since preliminary data indicate that the extent of linkage disequilibrium in cattle breeds is only slightly larger than in humans, it has been estimated that up to 300,000 SNPs will be necessary to achieve optimal marker coverage throughout the cattle genome [5-8].

Circumventing any pooling or enrichment protocols, we sequenced just a single Fleckvieh animal to identify a large number of candidate SNPs. We demonstrate that this approach represents an effective strategy towards a comprehensive resource for common SNPs.

## Results and Discussion

### Sequencing and alignment

**Figure 1:**
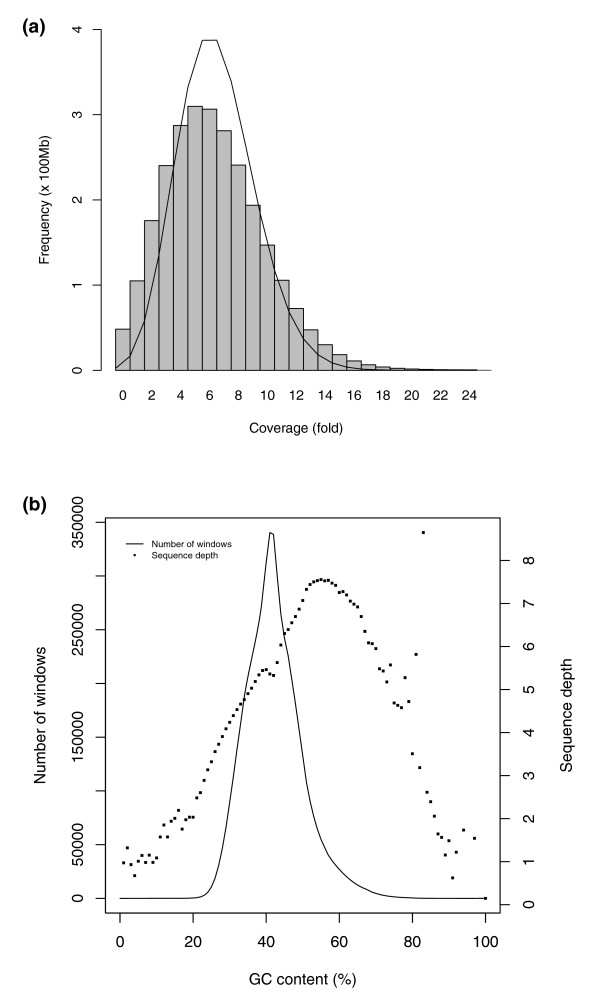
Distribution of read depth. **(a) **Distribution of mapped read depth in all autosomal chromosomes. Read depth is sampled at every position along the chromosomes. The solid line represents a Poisson distribution with the same mean. **(b) **Distribution of read depth as a function of GC-content. GC-content and read depth were calculated for non-overlapping windows of 500 bp.

The genomic DNA sequenced in this study was obtained from a single blood sample of a Fleckvieh breeding bull. Whole-genome sequencing was performed on an Illumina Genome Analyzer II using three different small-insert paired-end libraries. We generated 36-bp reads on 44 paired-end lanes and 9 single-end lanes, resulting in 24 Gb of mappable sequence. Of the aligned bases, 87% had a phred-like quality score of 20 or more, as calculated by the ELAND alignment software [9]. To account for the varying read quality, we trimmed the ends of reads when necessary to a minimum of 32 bases. Read mapping, subsequent assembly and SNP calling were performed using the re-sequencing software MAQ [10]. Apparently duplicated paired-end reads (7.6%) were removed. Of the paired-end reads, 605,630,585 (93.6%) were successfully mapped in mate-pairs to the assembly bosTau4.0 from October 2007 [11], which has a length of 2.73 Gb. Additionally, 23,872,053 of paired-end reads (3.6%) were mapped as singles. Of the 25,808,311 single-end reads, 93.2% could be aligned to the genome. Together, 98.0% of the genome (98.1% of the autosomes and 93.9% of the X chromosome) was covered by reads resulting in a 7.4-fold coverage across the entire genome (7.58-fold across the autosomes and 4.13-fold across the X chromosome) and a 6.2-fold sequence depth using only the uniquely aligned reads. The final distribution of mapped read depth sampled at every position of the autosomal chromosomes showed a slight over-dispersion compared to the Poisson distribution giving the theoretical minimum (Figure 1a). Part of this over-dispersion can be accounted for by the dependence of the read depth on the GC-content, which had a maximum average read depth at approximately 57% GC-content (Figure 1b) [9,12].

### SNP and indel detection

**Figure 2:**
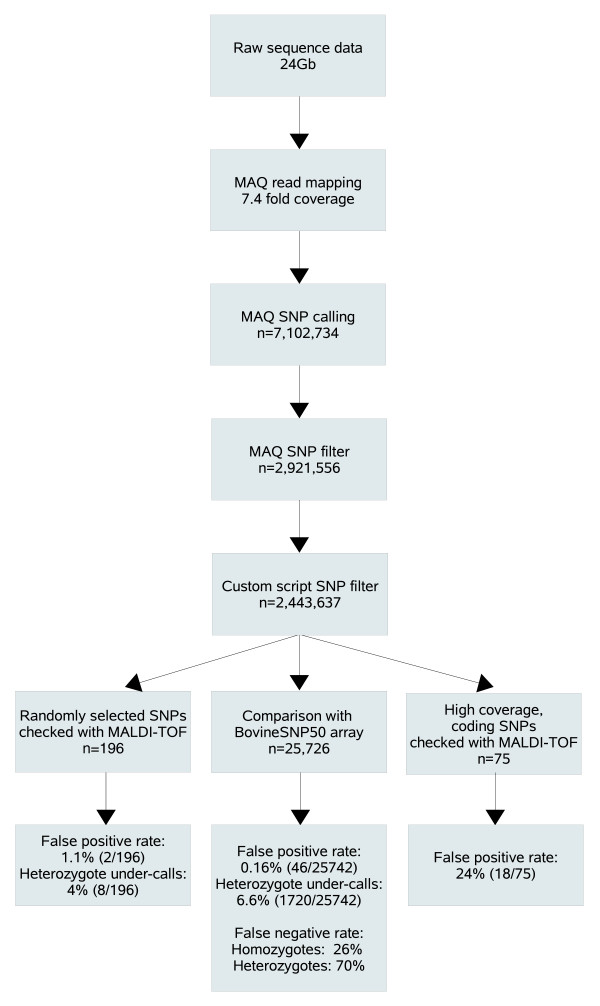
Analysis procedure. Sequence reads were aligned to the reference sequence (bosTau4) by the MAQ software. SNPs were called and filtered by MAQ and custom scripts, resulting in a final set of 2.44 million SNPs. Comparison with 25,726 array-based genotpyes revealed a false-negative detection rate of 49%. A false-positive detection rate of 1.1% was determined by comparison with 196 randomly selected SNPs genotyped with MALDI-TOF spectroscopy. By determining the false-positive detection rate in 75 coding SNPs with high coverage (≥16), we found evidence that the high false-positive detection rate in these SNPs is due to mapping errors caused by duplications that are not reflected in the reference sequence rather than to sequencing errors.

We focused our further analysis on SNP identification. We applied stringent criteria in order to keep the false-positive detection rate low. An outline of the analysis procedure, comprising SNP identification and validation, is given in Figure 2. SNPs were called with the MAQ software. Using mainly the default parameters, particularly a minimum read depth of 3 and a minimum consensus quality of 20, SNPs could be assessed in sequence reads, which together comprised 68% (1.87 Gb) of the genome. To exclude sequencing artifacts that we have observed in other experiments, the output of MAQ was further filtered using custom developed scripts. These artifacts include cases where all sequenced variant alleles at a given position are only indicated by reads from one strand and have a lower than average base quality at the variant position. We required for a SNP call that the average base quality is ≥20 and that at least 20% of the reads are from opposite strands. Using these parameters, the MAQ software called 2,921,556 million putative SNPs, which were reduced by our custom filters to a final set of 2.44 million SNPs.

Of these SNPs, 1,694,546 (69.4%) were homozygous and 749,091 (30.6%) were heterozygous. The low proportion of heterozygous SNPs is mainly due to the relatively low sequence depth and our stringent SNP calling requirements. The rate of heterozygous SNP detection is expected to rise with increasing coverage (Additional data file 1). It has been estimated that at least 20- to 30-fold coverage is needed to detect 99% of the heterozygous variants [10].

**Figure 3:**
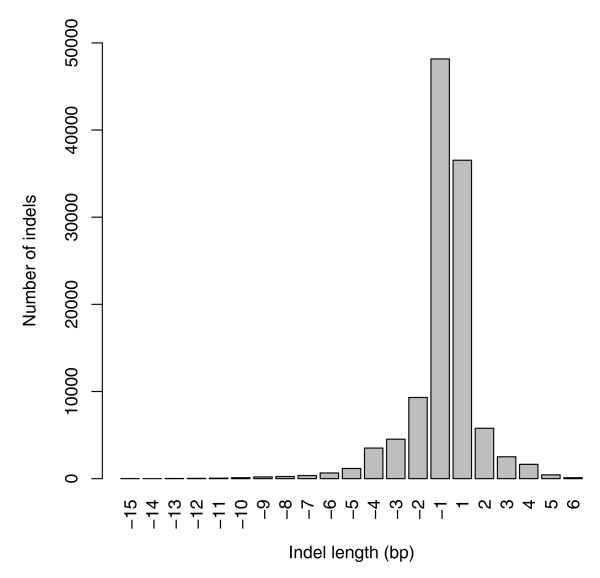
Small indels. Distribution of the size of 115,371 small indels (68,354 deletions and 47,017 insertions). Positive and negative values on the x-axis correspond to the presence or absence of bases relative to the reference sequence.

We further performed a genome-wide survey of small insertion and deletion events (indels). Indels called by MAQ were only retained if they were indicated by at least 10% of high-quality reads from each strand. This criterion was applied to exclude possible sequencing artifacts and resulted in the identification of 115,371 indels (68,354 deletions and 47,017 insertions). The majority of them had a length of 1 to 4 bp, with the largest having a length of 15 bp (Figure 3).

Next we compared the identified SNP and indel variants with those already published. Since the dbSNP set is not yet mapped to the bosTau4 assembly, we compared our findings with the 2.08 million SNPs mapped by the Baylor College Bovine Genome Project. The comparison showed that 18% (451,914) of the SNPs were shared between both sets (Table 1).

**Table 1:** Identified SNPs and small indels

	All	Coding	Non-synonymous	Splice-site	UTR
SNPs (Ensembl)	2,443,637 (18%)	22,070 (18%)	9,360 (15%)	148 (14%)	8,114 (20%)
Indel (Ensembl)	115,371	425			
SNP (RefSeq)	2,443,637 (18%)	7,619 (18%)	3,139 (16%)	40 (15%)	6,292 (20%)
Indel (RefSeq)	115,371	203			

Proportion of SNPs that have been previously reported are given in parentheses. UTR, untranslated region.

### Functional annotation

We used the RefSeq (9,518 genes) and Ensembl (28,045 genes) gene sets to functionally annotate the detected variants (Table 1). Using the RefSeq genes as reference, we found 7,619 coding SNPs (3,139 leading to non-synonymous amino acid substitutions), 40 SNPs at canonical splice sites and 6,292 SNPs in untranslated regions. Additionally, 203 indels were located in coding regions, with almost all of them (201) causing a frame-shift in the corresponding gene. The remaining two indels comprise single amino acid deletions.

The Ensembl gene set is larger and includes also gene predictions. Thus, more variants are detected using this set. We identified 22,070 coding SNPs (9360 non-synonymous substitutions), 148 SNPs at donor or acceptor splice sites and 8114 SNPs in untranslated regions. Furthermore, we identified 425 indels in Ensembl annotated coding regions. Most of them (414) cause a frame-shift in the reading frame of the associated gene, 9 indels lead to single amino acid deletions and 2 were single amino acid insertions.

### Comparison of sequence and array results

We assessed the accuracy and completeness of the sequence-based SNP calls by comparing them with the genotypes of the same animal generated with an Illumina BovineSNP50 array. This chip contains 54,001 SNPs, of which 48,188 map to the current assembly (bosTau4). Of those, 48,025 SNPs were successfully genotyped; 22,299 homozygous calls exhibited the reference allele, leaving 12,043 homozygous and 13,683 heterozygous SNPs that were different with respect to the reference sequence assembly. We used these 25,726 positions together with 16 positions where only the MAQ call differed from the reference sequence to examine the accuracy and sensitivity of SNP calling in more detail.

We first estimated the proportion of concordant calls. Of the 12,043 homozygous array-based calls that differed from the reference sequence, 8,974 (74.51%) were also called by MAQ. In 8,949 (99.72%) of these positions, both platforms showed concordant genotypes. Of the 13,683 heterozygous array-based calls, MAQ called only 5,882 (42.98%) positions, and only 4,157 (70.67%) of these matched the array results (Table 2). The false-negative rate of sequenced SNPs as judged from the array experiment is therefore 26% (100 - 8,949/12,043) for the homozygous variants and 70% (100 - 4,157/13,683) for the heterozygous genotypes. Based on these estimates, the investigated genome contains 2,289,927 homozygous and 2,496,970 heterozygous SNPs. The combined false-negative rate would be 49% (100 - (8,949 + 4,157)/(12,043 + 13,683)), which is more than expected from simulation studies at a sequence depth of 6 to 7.4 [10].

**Table 2:** Concordant calls

	BovineSNP50	MAQ calls	Concordant calls
Homozygote reference	22,999		
Homozygote variant	12,043	8,974 (74.51%)	8,949 (99.72%)
Heterozygote	13,683	5,882 (42.98%)	4,157 (70.67%)

Comparison of the SNP calls made from genotype data and the sequence: concordant calls. Genotype data were generated using the Infinium BovineSNP50 BeadChip. Homozygote reference denotes an array-based genotype that is homozygous for the reference allele. Homozygote variant denotes an array-based genotype that is homozygous for a non-reference allele. Heterozygote denotes a heterozygous array-based genotype containing one reference allele and a variant allele.

We then determined the disagreements in more detail, which are composed of the 1,750 discordant calls plus the 16 positions where MAQ called a SNP while the genotyping result was identical to the reference sequence (Table 3). Of the 1,766 disagreements, 1,720 were heterozygote under-calls of MAQ. 'Heterozygote under-call' denotes a homozygous sequencing SNP at the position of a heterozygous genotyping SNP where the sequencing SNP corresponds to one of the two heterozygous genotyping alleles. For 10 of the remaining 46 differing positions, a heterozygote call was made by MAQ whereas the genotyping array only showed the reference allele, indicating a possible heterozygote under-call by the array. At one of these positions the array tests for a different variant allele than the one detected by MAQ (chip result CC, chip test alleles CT, MAQ CG, reference C). At 15 positions the platforms showed different homozygous genotypes that both differed from the reference genotype. At 21 positions we observed other differences. Assuming that these 46 SNPs are wrong calls, the false-positive rate would therefore be 0.16% (46 out of 25,742).

**Table 3:** Discordant calls

	Discordant calls
All disagreements	1,766 (6.86%)
GT-het>Seq-hom	1,720 (6.68%)
Seq-het>GT-hom	10 (0.03%)
Different homozygotes	15 (0.06%)
Different heterozygotes	5 (0.02%)
Seq-SNP>GT-Ref	16 (0.09%)

Comparison of the SNP calls made from genotype data and the sequence: discordant calls. GT-het>Seq-hom indicates a heterozygote under-call by MAQ (array based genotype heterozygote, MAQ based genotype homozygote). Seq-het>GT-hom indicates a possible heterozygote under-call by the array (array-based genotype homozygote for the reference allele, MAQ based genotype heterozygote). Different homozygotes denote homozygous genotypes on both platforms that both differed from the reference genotype. Different heterozygotes denote heterozygote genotypes on both platforms where one allele differs. Seq-SNP>GT-Ref indicates a MAQ based genotype that differs from the reference sequence while the chip based genotype displayed only the reference allele.

We also estimated the autosomal nucleotide diversity π taking into account that we identified only 30% of the heterozygous SNPs correctly. This led to an autosomal nucleotide diversity of approximately 9.4 × 10^-4 ^or 1 SNP per 1,060 bp ((749,091 - 3,553)/0.30/(2.73e9 - 88,000,000) [(Heterozygous_SNPs - X_chromosomal_SNPs)/Detection_rate/(Genome_length - X_chromosome_length)]). This value is higher than the nucleotide diversity observed in humans [9,13] but in accordance with previous estimates in Fleckvieh [14,15]. To assess the nucleotide diversity in coding regions, we constructed a non-redundant gene set based on the Ensembl genes by merging all transcripts from the same gene into a single 'maximum coding sequence', resulting in 22,796 non-redundant genes. According to this set, the total coding sequence length for cattle is 33,235,846 bp, or 1.21% of the genome. This coding region contained 8,438 heterozygous SNPs, resulting in a nucleotide diversity of 8.5 × 10^-4 ^or 1 SNP per 1,181 bp (8,438/0.30/(33,235,846)).

### SNP genotyping

To further evaluate the false-positive discovery rate of SNP calling, we randomly selected a subset of 104 homozygous and 104 heterozygous SNPs from genomic regions, defined by uniquely aligned reads, and genotyped them using multiplex MALDI-TOF (matrix-assisted laser desorption/ionization time-of-flight) mass spectrometry. Contigs that were not allocated to a specific chromosome were excluded. The distribution of read depth of the selected SNPs was similar to that of the entire SNP set (Additional data file 2). To enable design of the extension primer, we did not allow for other SNPs to occur 20 bp upstream and downstream of the target SNP. In addition, we masked all other SNPs in the 200-bp fragment used for the design of the amplification primers. Genotypes could be successfully determined for 196 assays, with an average call rate of 98.3% (Table 4). We detected ten disagreements, eight of which were heterozygous sequencing under-calls, which were not considered for the calculations. These undercalls are expected due to inadequate sampling of alleles when sequencing at a fairly low coverage level. On that basis, the false-positive discovery rate was calculated to be 1.1% (2 of 186).

**Table 4:** SNPs called by MAQ compared with calls by MALDI-TOF genotyping

Concordant calls	186
MAQ heterozygote under-call	8
MALDI-TOF homozygous, MAQ heterozygous	2
Error rate (without heterozygote under-calls)	1.1%

**Figure 4:**
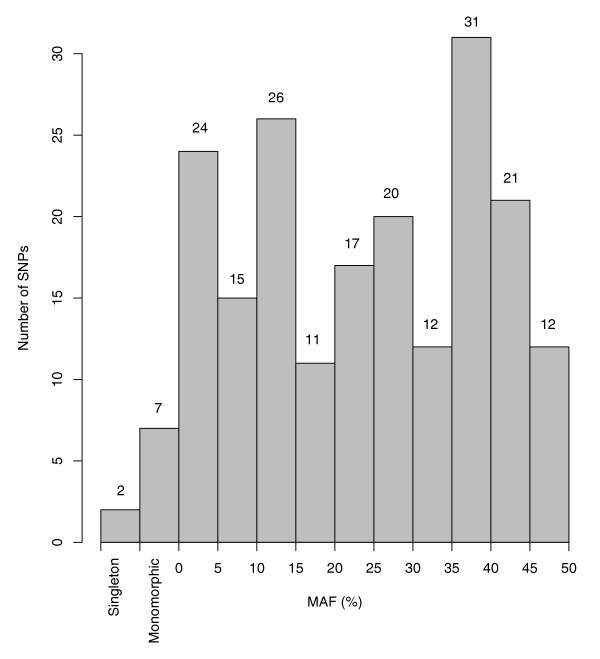
Minor allele frequency (MAF) spectrum of randomly selected SNPs. Genotypes of 196 SNPs were determined by MALDI-TOF mass spectroscopy in 48 Fleckvieh and 48 Braunvieh bulls.

To estimate the population frequencies, we assayed the same SNPs in 48 Braunvieh and 48 Fleckvieh bulls that were selected to be not closely related (Additional data file 3). Two SNPs turned out to be singletons only present in the bull that had been sequenced and seven were monomorphic for the variant allele. The mean MAF of the remaining 187 SNPs was 24.5%. The distribution of the minor allele frequency of tested SNPs was nearly uniform (Figure 4) [16]. The distribution shows that 83% of the SNPs had a MAF of 5% or more, which makes them suitable for association studies using common SNPs in these breeds.

In an attempt to select SNPs specifically from coding regions, we selected 75 SNPs only from regions with high sequence depth (≥16) under the assumption that sensitivity and specificity should gain from higher coverage. Because only 5.8% of coding SNPs had a sequence depth of 16 or more, several SNPs were located in close proximity. Contrary to our expectation, comparison with MALDI-TOF genotypes resulted in a false-positive rate as high as 24% (18 of 75). All these SNPs were called as heterozygotes by MAQ. Of these SNPs, 11 were called as homozygotes by MALDI-TOF genotyping in all 96 investigated animals. The remaining 7 were counted as false-positives because they were called as heterozygotes by MALDI-TOF genotyping in all 96 investigated animals. These sites were also ambiguous when checked by capillary sequencing in 12 selected animals (Additional data file 4). We therefore suspected that the selection from the extreme of coverage has introduced a strong bias. The false-positive calls were most likely caused by reads that were misassembled because these regions are duplicated but only one copy is contained in the reference sequence. Checking the read depth around the false-positive SNPs, we found 3 SNPs (chr4_117247234, chr4_117247581, chr13_16920248) that were obviously located in regions of 30 and 300 kb with high average read depth, indicating a duplication of that region (Additional data file 5). In the other regions, the high read depth extended only across a short distance so that we can not exclude random noise. It was further noticeable that several of the false-positive SNPs were located near gaps or in regions with several gaps, suggesting assembly difficulties. Although we can not provide an unequivocal explanation for the high false-positive rate of SNPs in regions with high read depth, we want to point out that these errors do not compromise the overall false-positive detection rate of 1.1%. Rather, it reveals that a significant proportion of heterozygous false-positives are not caused by sequencing errors but, most likely, by erroneous alignment and that the risk for this type of error is negatively correlated with the quality and completeness of the reference sequence. This information can be used to further filter the SNP set. Discarding all SNPs with a read depth ≥16 would reduce the set by 53,259 SNPs (2.2%).

## Conclusions

By sequencing a single diploid genome to a depth of 7.4-fold, we were able to generate more than 2 million SNPs, thereby almost doubling the existing SNP resource in cattle. We evaluated the error rates of SNP detection in detail, point out possible sources of errors and propose means for filtering error-prone SNPs. We deduced an overall false-positive detection rate of 1.1% from genotyping 196 randomly selected SNPs by an alternative technique. This value compares well with the reported false-positive detection rate of 2.5% estimated by genotyping 1,206 SNPs by a similar approach [9]. Despite a false-negative detection rate of 49%, which is largely explained by missing heterozygous SNPs at low sequencing coverage, SNP identification was very effective. In contrast to the detection of SNPs and small indels, the identification of structural variations at a size that exceeds the individual read length was ineffective at low sequence depth. In addition to SNP discovery, this sequence of a single animal constitutes a first step towards a haplotype reconstruction of the Fleckvieh breed. The animal selected for this approach was a prominent Bavarian Fleckvieh bull. With more than 50,000 inseminations in 2008 alone, the selected animal is founder of a very large pedigree. Fleckvieh is a dual purpose breed (dairy and beef) originating from the Swiss Simmental breed. Fleckvieh cows contribute about 8% of all recorded lactations worldwide, which makes them the second largest dairy breed after Holstein. Fleckvieh, together with the Brown breed, are so called Alpine breeds that are phylogenetically distant from Holstein [17]. The distribution of genotypes found for 196 SNPs in 48 Brown and 48 Fleckvieh animals proved our chosen strategy to be successful. We provide a comprehensive SNP list for the two main Alpine breeds Brown and Fleckvieh. For a future dense array with up to 1 million SNPs, the experiment provides SNPs that can be translated into genome-wide oligonucleotide arrays in a single-step procedure with a conversion rate of more than 80%. The chosen strategy is predicted to be applicable to complement the SNP resource in other farm animals such as swine and chicken, especially with sequencing outputs from a single experiment predicted to cross the 100 Gb threshold before the end of 2009.

## Materials and methods

### DNA library construction and sequencing

EDTA-blood was obtained from Fleckvieh bull Vanstein 191658 and genomic DNA was extracted according to standard protocols. DNA was sheared by nebulization with compressed nitrogen gas. We constructed 3 different paired-end libraries with median insert sizes of 75, 80 and 170 nucleotides. The libraries were sequenced on a GAII (Illumina, San Diego, Californica, USA). Sample preparation, cluster generation and sequencing were performed according to the manufacture's protocols with minor modifications (Illumina paired-end cluster generation kit GA II v1, 36-cycle sequencing kit v1).

### Analysis software

We used the bosTau4.0 assembly as reference sequence including the scaffolds that were not anchored onto specific chromosomes. Image analysis and ELAND alignment was performed with the Pipeline software version 1.0 as provided by Illumina. Subsequently, short read alignment, consensus assembly and variant calling were performed using the re-sequencing software MAQ version 0.6.8 [10]. For the alignment part, we used the following parameters: number of maximum mismatches that can always be found = 2; mutation rate between the reference sequence and the reads = 0.001; threshold on the sum of mismatching base qualities = 70. For the 'snpfilter' part of the MAQ software, we used the following parameters: minimum read depth = 3; maximum read depth = 256; minimum mapping quality = 40; minimum neighboring quality = 20; minimum consensus quality = 20; window size around potential indels = 3; window size for filtering dense SNPs = 10; maximum number of SNPs in a window = 2.

After SNP calling by MAQ, we applied additional filters. We required each putative SNP to have a median quality value of the variant base of at least 20 and that at least 20% of the reads covering this position must come from opposite strands. Functional analysis of the SNPs was performed with custom Perl scripts using datasets from Ensembl [18], the Santa Cruz Genome Browser [19] and the Baylor College Bovine Genome Project web pages [20]. Ensembl and RefSeq gene annotations were used as provided by the Santa Cruz Genome Browser (October 2008). SNP locations were downloaded form the Baylor College Bovine Genome Project ftp site [21].

### Genotyping

For genotyping, we selected bulls that did not have both sires and maternal grandsires in common. Genotypes were determined on a BovineSNP50 chip (Illumina). Genotyping of selected SNPs was performed with the MassARRAY system (Sequenom, San Diego, California, USA) using the iPLEX Gold chemistry. For random selection of SNPs we used a random number generator as implemented in the Perl function 'rand'. Assays were designed using AssayDesign 3.1.2.2 with iPLEX Gold default parameters and up to 25 assays were multiplexed. Genotype calling was done with SpectroTYPER 3.4 software.

### Data access

Sequence data are available from the European Read Archive (ERA) [ERA:ERA000089]. SNPs have been submitted to dbSNP ([dbSNP:ss140006985] to [dbSNP:ss142339932]).

## Abbreviations

Indel: small insertion/deletion event; MAF: minor allele frequency; MALDI-TOF: matrix-assisted laser desorption/ionization time-of-flight; SNP: single nucleotide polymorphism.

## Authors' contributions

RF, TM and TMS conceived of the study and participated in its design and coordination. ABP with KF performed the sequencing experiments. SHE and TMS performed the data analysis. SHE, TM, RF and TMS drafted the manuscript. All authors contributed to and approved the final manuscript.

## Additional data files

The following additional data are available with the online version of this paper: a table showing the number of homo- and heterozygous SNPs depending on different read depth (Additional data file 1); a figure showing empirical cumulative distribution of read depth of the SNPs selected for MALDI-TOF genotyping in comparison to the entire SNP set (Additional data file 2); a table showing genotypes, MAF and test for Hardy-Weinberg equilibrium of 196 SNPs determined with MALDI-TOF spectroscopy in 48 Fleckvieh and 48 Braunvieh bulls (Additional data file 3); a table showing the false-positive SNP calls in 75 coding SNPs with high read depth (≥16) (Additional data file 4); a figure showing the sequencing depth around false-positive MAQ calls (Additional data file 5).

## Supplementary Material

Additional data file 1 Number of homo- and heterozygous SNPs depending on different read depth. Click here for file

Additional data file 2 Empirical cumulative distribution of read depth of the SNPs selected for MALDI-TOF genotyping in comparison to the entire SNP set. Click here for file

Additional data file 3 Testing of Hardy-Weinberg equilibrium was performed with Pearson's goodness-of-fit chi-square test with one degree of freedom. Click here for file

Additional data file 4 Calls by MAQ were checked by MALDI-TOF spectroscopy and capillary sequencing. Click here for file

Additional data file 5 Part of the false-positive calls are obviously located in regions with higher than average read depth, suggesting duplications. Read depth in approximately unique regions was calculated for non-overlapping windows of 500 bp by the MAQ software. SNP calls are indicated by grey vertical lines. The identifier of the SNP is indicated in the figure legend. Click here for file
