# Supplementary material for: Whole genome sequencing of a single Bos taurus animal for single nucleotide polymorphism discovery
Source: Genome Biol. 2009 Aug 6;10(8):R82. doi: 10.1186/gb-2009-10-8-r82 (PMC2745763; doi:10.1186/gb-2009-10-8-r82)
Supplement: Additional data file 1 — Number of homo- and heterozygous SNPs depending on different read depth. [file gb-2009-10-8-r82-S1.pdf]

Additional file 1. Number of homo- and heterozygous SNPs depending on different read depth.

	Homozygotes	Heterozygotes
All	1,694,546 (69.34%)	749,091 (30.66%)
Read depth ≥ 5	1,302,059 (63.48%)	749,091 (36.52%)
Read depth ≥ 10	287,252 (47.34%)	319,531 (52.66%)
Read depth ≥ 15	26,448 (33.83%)	51,720 (66.16%)
