# Supplementary figures and images for: Whole genome sequencing of a single Bos taurus animal for single nucleotide polymorphism discovery
Source: Genome Biol. 2009 Aug 6;10(8):R82. doi: 10.1186/gb-2009-10-8-r82 (PMC2745763; doi:10.1186/gb-2009-10-8-r82)

### Homozygotes

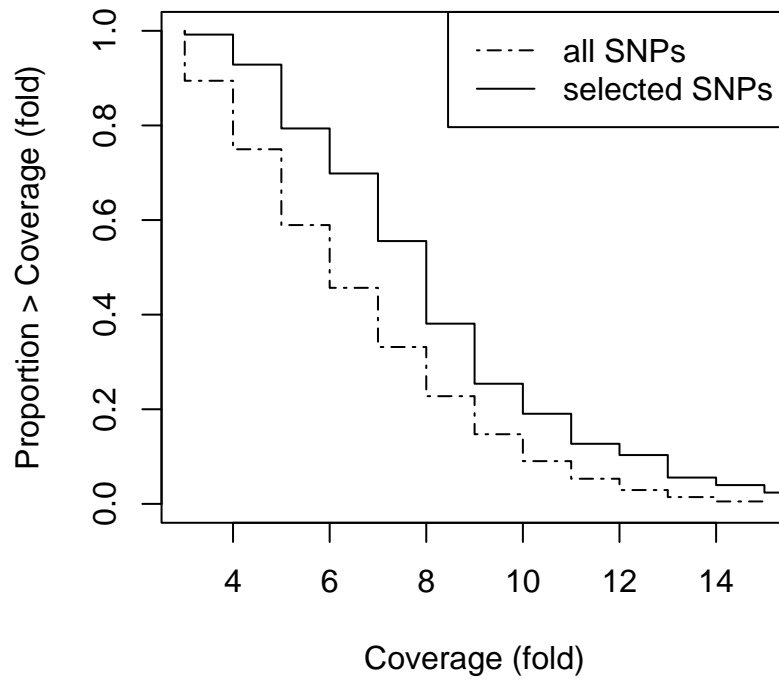

### Heterozygotes

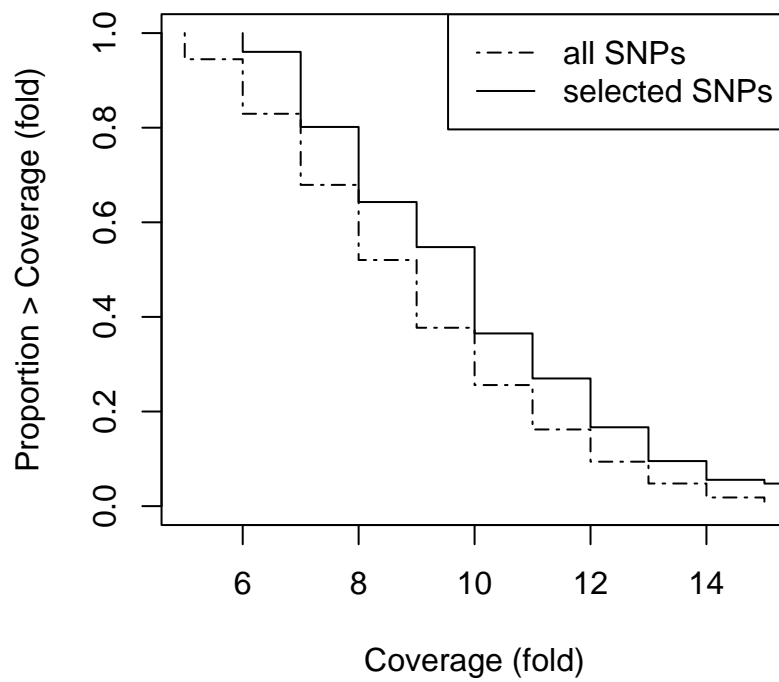

Supplement: Additional data file 2 — Empirical cumulative distribution of read depth of the SNPs selected for MALDI-TOF genotyping in comparison to the entire SNP set. [file gb-2009-10-8-r82-S2.pdf]

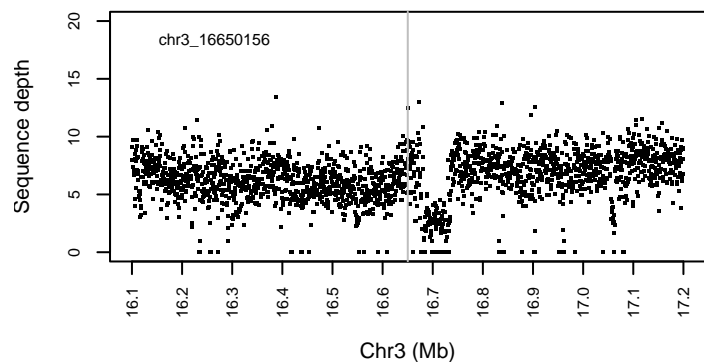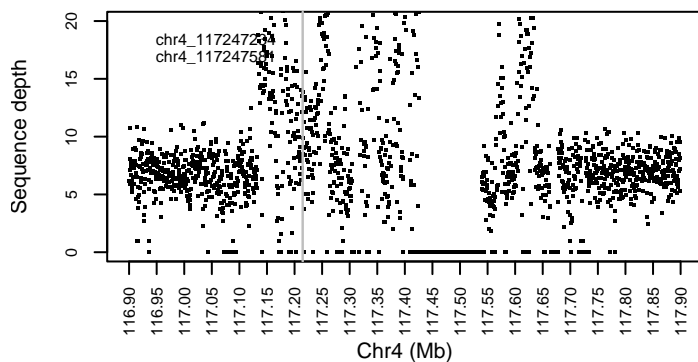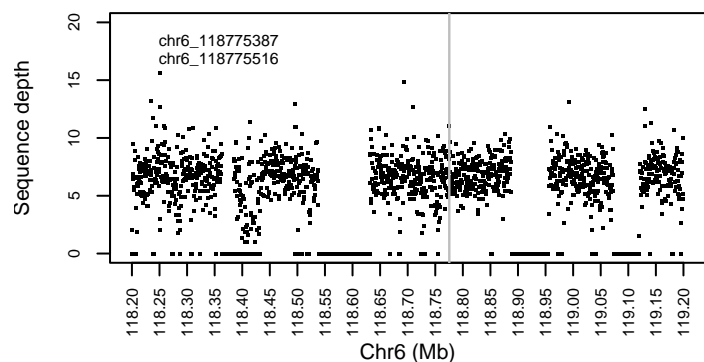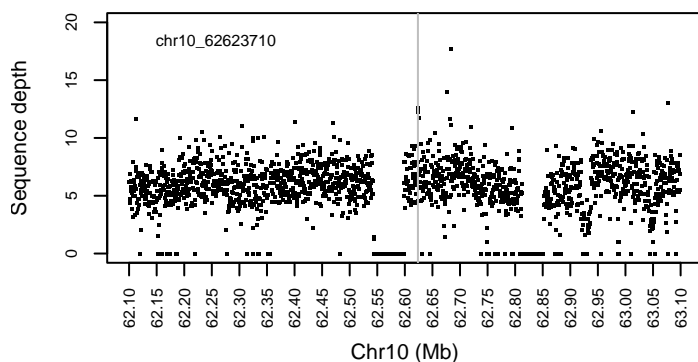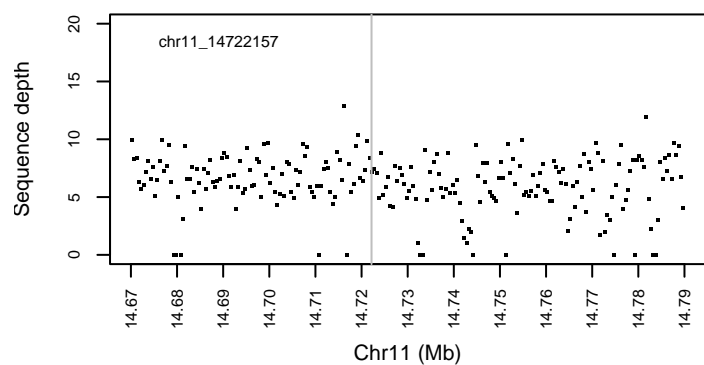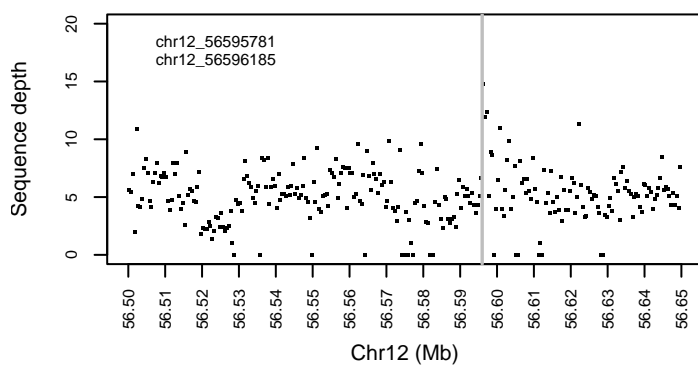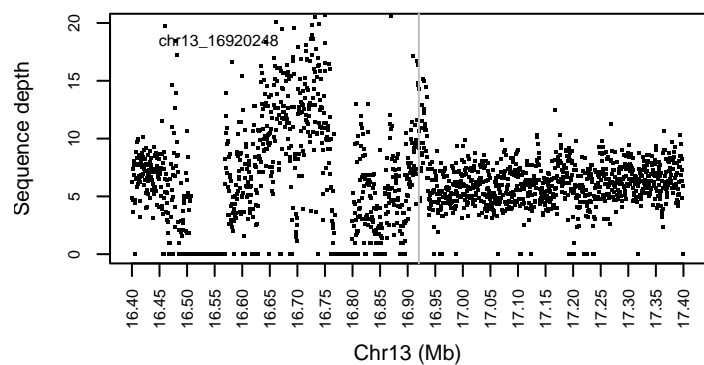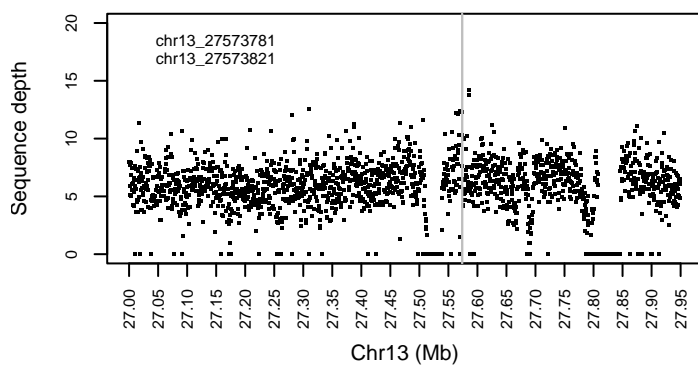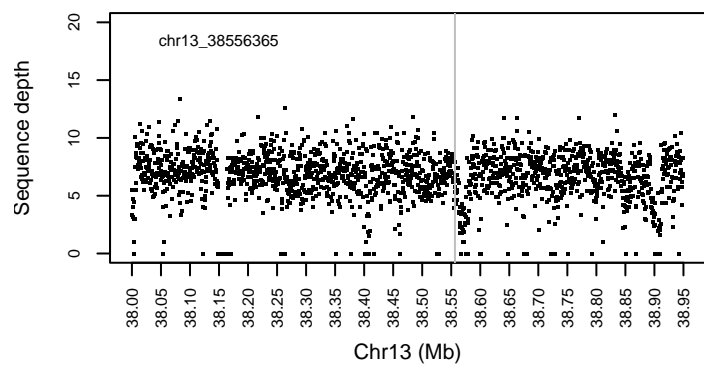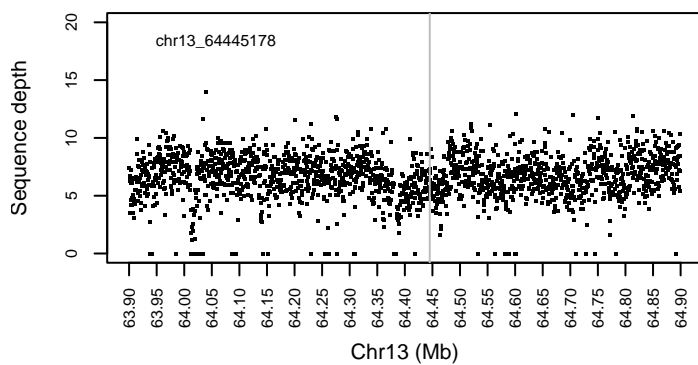

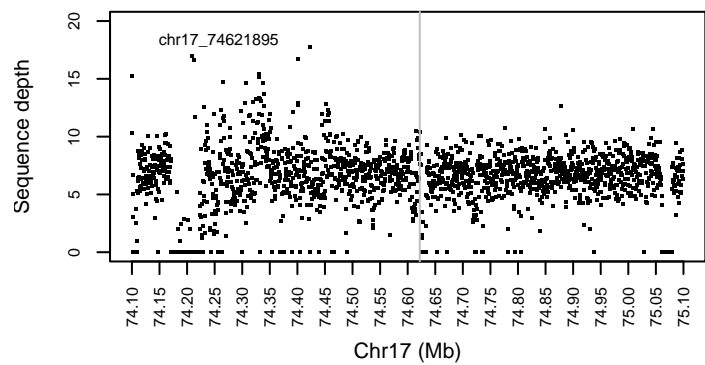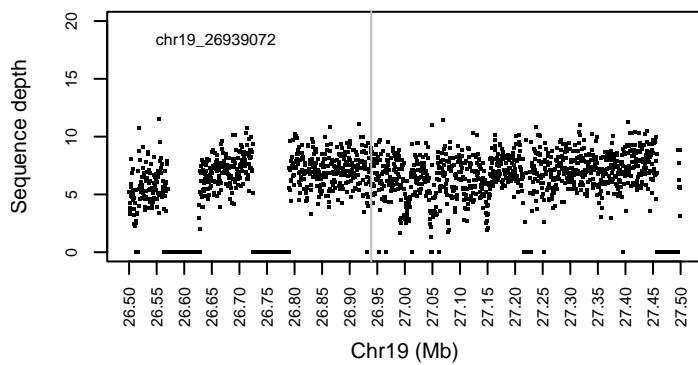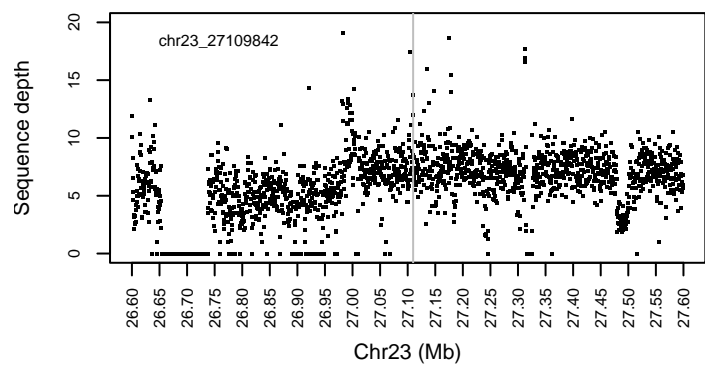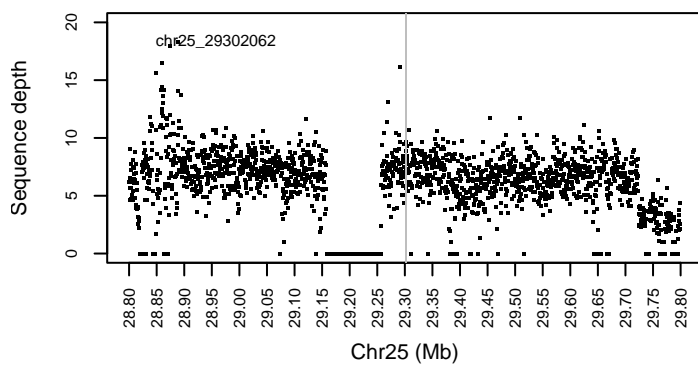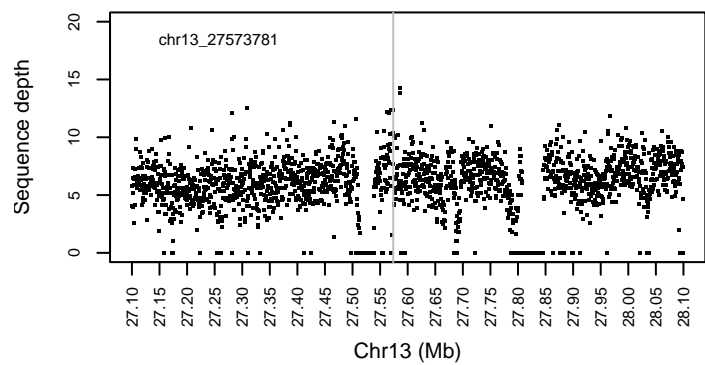

Supplement: Additional data file 5 — Part of the false-positive calls are obviously located in regions with higher than average read depth, suggesting duplications. Read depth in approximately unique regions was calculated for non-overlapping windows of 500 bp by the MAQ software. SNP calls are indicated by grey vertical lines. The identifier of the SNP is indicated in the figure legend. [file gb-2009-10-8-r82-S5.pdf]
