# Supplementary material for: Whole genome sequencing of a single Bos taurus animal for single nucleotide polymorphism discovery
Source: Genome Biol. 2009 Aug 6;10(8):R82. doi: 10.1186/gb-2009-10-8-r82 (PMC2745763; doi:10.1186/gb-2009-10-8-r82)
Supplement: Additional data file 3 — Testing of Hardy-Weinberg equilibrium was performed with Pearson's goodness-of-fit chi-square test with one degree of freedom. [file gb-2009-10-8-r82-S3.pdf]

Additional file 3. Genotypes of 196 SNPs determined with MALDI-TOF spectroscopy in 48 Fleckvieh and 48 Braunvieh bulls.

SNP	Called genotypes (percent)						MAF	Hardy-Weinberg (Pearson)
chr1_100019362	CC	91 (91.02)	CT	3 (2.95)	TT	0 (0.02)	0.02	0.87507
chr1_127825345	CC	5 (4.30)	CT	30 (31.40)	TT	58 (57.30)	0.22	0.667675
chr1_138224434	AA	87 (87.13)	GA	7 (6.74)	GG	0 (0.13)	0.04	0.707691
chr1_141095630	CC	34 (35.79)	CT	48 (44.43)	TT	12 (13.79)	0.38	0.43534
chr1_144127759	CC	16 (13.55)	CT	39 (43.90)	TT	38 (35.55)	0.38	0.281937
chr1_157787695	AA	18 (14.72)	AT	38 (44.56)	TT	37 (33.72)	0.40	0.155738
chr1_24135289	AA	18 (14.72)	AT	38 (44.56)	TT	37 (33.72)	monomorph	0.155738
chr1_3073018	AA	18 (17.64)	GA	45 (45.73)	GG	30 (29.64)	0.44	0.87834
chr1_35778643	AA	12 (12.80)	AG	45 (43.40)	GG	36 (36.80)	0.37	0.722752
chr1_474300	AA	12 (12.80)	AG	45 (43.40)	GG	36 (36.80)	monomorph	0.722752
chr1_64366278	AA	36 (34.94)	CA	42 (44.13)	CC	15 (13.94)	0.39	0.641743
chr1_74241064	CC	86 (77.85)	CT	0 (16.29)	TT	9 (0.85)	0.09	0.641743
chr1_84388572	AA	5 (2.87)	GA	23 (27.27)	GG	67 (64.87)	0.17	0.127085
chr1_98066301	GG	9 (10.22)	GT	44 (41.55)	TT	41 (42.22)	0.33	0.568068
chr10_16844660	AA	24 (25.02)	CA	49 (46.95)	CC	21 (22.02)	0.48	0.672387
chr10_6241614	CC	81 (81.39)	CT	12 (11.23)	TT	0 (0.39)	0.06	0.506
chr10_64092955	AA	39 (42.68)	AG	48 (40.65)	GG	6 (9.68)	0.32	0.080978
chr10_73226822	CC	77 (76.78)	TC	15 (15.45)	TT	1 (0.78)	0.09	0.78055
chr10_83477556	AA	70 (70.78)	GA	24 (22.44)	GG	1 (1.78)	0.14	0.498656
chr10_88800991	AA	4 (5.63)	CA	38 (34.74)	CC	52 (53.63)	0.24	0.363676
chr10_92110206	CC	14 (12.30)	CT	40 (43.40)	TT	40 (38.30)	0.36	0.447003
chr11_107889496	AA	2 (0.52)	GA	10 (12.96)	GG	82 (80.52)	0.07	0.447003
chr11_19805849	AA	58 (58.09)	AT	31 (30.82)	TT	4 (4.09)	0.21	0.955732
chr11_20626618	CC	33 (32.77)	CT	45 (45.46)	TT	16 (15.77)	0.41	0.921385
chr11_28772929	CC	43 (37.66)	CT	33 (43.68)	TT	18 (12.66)	0.37	0.921385
chr11_46420781	AA	37 (36.80)	GA	43 (43.40)	GG	13 (12.80)	0.37	0.928612
chr11_76430734	AA	16 (16.18)	AG	46 (45.64)	GG	32 (32.18)	0.41	0.938751
chr11_85715088	AA	29 (20.60)	AT	30 (46.81)	TT	35 (26.60)	0.47	0.938751

chr12_12056372	AA	74 (74.08)	GA	18 (17.85)	GG	1 (1.08)	0.11	0.935178
chr12_20002871	GG	93 (93.00)	GA	1 (0.99)	AA	0 (0.00)	0.01	0.958651
chr12_44193880	CC	34 (33.36)	CT	44 (45.28)	TT	16 (15.36)	0.40	0.784572
chr12_9684670	AA	38 (35.79)	GA	40 (44.43)	GG	16 (13.79)	0.38	0.334134
chr13_17956577	AA	60 (61.60)	GA	33 (29.79)	GG	2 (3.60)	0.19	0.294389
chr13_26147424	AA	9 (5.20)	GA	26 (33.59)	GG	58 (54.20)	0.24	0.294389
chr13_41192630	CC	4 (0.32)	CT	3 (10.36)	TT	87 (83.32)	0.06	0.294389
chr13_55018386	GG	57 (54.97)	GT	29 (33.06)	TT	7 (4.97)	0.23	0.236378
chr13_55250521	CC	55 (53.63)	CT	32 (34.74)	TT	7 (5.63)	0.24	0.443741
chr13_62528973	AA	65 (63.75)	AG	24 (26.49)	GG	4 (2.75)	0.17	0.363875
chr13_65919379	CC	18 (14.17)	TC	37 (44.65)	TT	39 (35.17)	0.39	0.096533
chr13_66007548	CC	14 (11.94)	TC	39 (43.12)	TT	41 (38.94)	0.36	0.354009
chr13_80890372	AA	2 (3.68)	GA	33 (29.64)	GG	58 (59.68)	0.20	0.274269
chr14_24621053	AA	35 (36.02)	AG	47 (44.95)	GG	13 (14.02)	0.38	0.657102
chr14_3616069	CC	92 (92.01)	CT	2 (1.98)	TT	0 (0.01)	0.01	0.91697
chr14_5599126	CC	36 (37.66)	CT	47 (43.68)	TT	11 (12.66)	0.37	0.460523
chr14_58340743	AA	47 (46.84)	AG	38 (38.32)	GG	8 (7.84)	0.29	0.935302
chr14_60653998	CC	2 (1.19)	CG	17 (18.63)	GG	74 (73.19)	0.11	0.399062
chr14_60749788	GG	50 (47.76)	GT	34 (38.49)	TT	10 (7.76)	0.29	0.258115
chr15_13415086	CC	19 (19.42)	CT	47 (46.16)	TT	27 (27.42)	0.46	0.860011
chr15_17346376	AA	17 (16.35)	GA	44 (45.29)	GG	32 (31.35)	0.42	0.78351
chr15_20616465	AA	4 (3.26)	AG	27 (28.48)	GG	63 (62.26)	0.19	0.613463
chr15_2565415	CC	35 (31.11)	TC	37 (44.78)	TT	20 (16.11)	0.42	0.095726
chr15_26608136	GG	88 (88.10)	GA	6 (5.81)	AA	0 (0.10)	0.03	0.749251
chr15_57571861	AA	19 (17.45)	GA	43 (46.10)	GG	32 (30.45)	0.43	0.514289
chr15_67074394	CC	32 (30.45)	GC	43 (46.10)	GG	19 (17.45)	0.43	0.514289
chr15_67506992	AA	69 (70.55)	CA	24 (20.90)	CC	0 (1.55)	0.13	0.153094
chr15_76978304	GG	50 (50.65)	GT	38 (36.70)	TT	6 (6.65)	0.27	0.731711
chr15_8115940	AA	25 (21.29)	AG	39 (46.41)	GG	29 (25.29)	0.48	0.123454
chr16_19112443	AA	5 (4.35)	GA	30 (31.30)	GG	57 (56.35)	0.22	0.689412
chr16_5753844	CC	82 (82.27)	CT	10 (9.46)	TT	0 (0.27)	0.05	0.581466

chr16_60509655	AA	10 (12.07)	CA	47 (42.87)	CC	36 (38.07)	0.36	0.352301
chr16_61442047	CC	39 (39.59)	CT	44 (42.83)	TT	11 (11.59)	0.35	0.791086
chr16_63639372	AA	86 (86.17)	CA	8 (7.66)	CC	0 (0.17)	0.04	0.666538
chr16_66098641	AA	19 (17.64)	AG	43 (45.73)	GG	31 (29.64)	0.44	0.565374
chr17_12716096	CC	13 (11.94)	CT	41 (43.12)	TT	40 (38.94)	0.36	0.633238
chr17_22183186	CC	13 (11.94)	CT	41 (43.12)	TT	40 (38.94)	monomorph	0.633238
chr17_27994191	AA	5 (6.92)	GA	41 (37.16)	GG	48 (49.92)	0.27	0.317078
chr17_65936503	AA	12 (10.12)	GA	38 (41.77)	GG	45 (43.12)	0.33	0.379199
chr17_69634290	AA	51 (48.99)	AG	33 (37.02)	GG	9 (6.99)	0.27	0.29542
chr18_3818756	AA	1 (0.52)	GA	12 (12.96)	GG	81 (80.52)	0.07	0.473741
chr18_47436974	CC	13 (8.05)	GC	29 (38.91)	GG	52 (47.05)	0.29	0.473741
chr18_62383856	CC	12 (15.87)	CT	52 (44.26)	TT	27 (30.87)	0.42	0.095462
chr18_7186485	CC	85 (85.22)	CT	9 (8.57)	TT	0 (0.22)	0.05	0.625921
chr19_13909106	AA	52 (55.57)	TA	39 (31.86)	TT	1 (4.57)	0.22	0.625921
chr19_28497627	CC	8 (11.01)	CT	48 (41.98)	TT	37 (40.01)	0.34	0.16657
chr19_37894204	AA	2 (1.82)	GA	22 (22.37)	GG	69 (68.82)	0.14	0.874743
chr19_42970494	CC	9 (23.50)	CT	76 (47.00)	TT	9 (23.50)	0.50	0.874743
chr19_53094346	GG	87 (87.13)	GA	7 (6.74)	AA	0 (0.13)	0.04	0.707691
chr19_53479785	CC	9 (7.76)	CG	36 (38.49)	GG	49 (47.76)	0.29	0.530618
chr19_5513198	AA	48 (46.63)	AG	35 (37.73)	GG	9 (7.63)	0.29	0.487126
chr19_60748509	CC	81 (81.45)	GC	13 (12.10)	GG	0 (0.45)	0.07	0.471385
chr19_7600352	CC	41 (37.66)	CG	37 (43.68)	GG	16 (12.66)	0.37	0.138374
chr19_990682	AA	25 (25.04)	GA	46 (45.91)	GG	21 (21.04)	0.48	0.985506
chr2_107313890	AA	68 (69.80)	CA	26 (22.40)	CC	0 (1.80)	0.14	0.119697
chr2_118872651	AA	68 (69.80)	CA	26 (22.40)	CC	0 (1.80)	monomorph	0.119697
chr2_124464160	AA	68 (69.80)	CA	26 (22.40)	CC	0 (1.80)	monomorph	0.119697
chr2_27960485	CC	17 (10.22)	TC	28 (41.55)	TT	49 (42.22)	0.33	0.119697
chr2_63697794	AA	68 (68.09)	AG	24 (23.83)	GG	2 (2.09)	0.15	0.944789
chr2_82141093	CC	88 (88.10)	CA	6 (5.81)	AA	0 (0.10)	0.03	0.749251
chr20_13967136	AA	3 (1.20)	AG	15 (18.60)	GG	74 (72.20)	0.11	0.063196
chr20_32554041	AA	13 (11.48)	GA	39 (42.04)	GG	40 (38.48)	0.35	0.488197

chr20_44915469	AA	91 (91.01)	GA	2 (1.98)	GG	0 (0.01)	0.01	0.916517
chr20_46338672	AA	38 (37.66)	CA	43 (43.68)	CC	13 (12.66)	0.37	0.880797
chr20_57448776	AA	38 (37.66)	CA	43 (43.68)	CC	13 (12.66)	monomorph	0.880797
chr20_69028741	TT	90 (90.04)	CT	4 (3.91)	CC	0 (0.04)	0.02	0.833068
chr20_69280371	CC	36 (31.35)	CT	36 (45.29)	TT	21 (16.35)	0.42	0.833068
chr20_71533294	CC	2 (1.53)	TC	20 (20.94)	TT	72 (71.53)	0.13	0.664628
chr21_12169058	AA	59 (60.33)	CA	31 (28.34)	CC	2 (3.33)	0.19	0.368445
chr21_26623232	CC	68 (68.94)	CT	25 (23.12)	TT	1 (1.94)	0.14	0.431097
chr21_28996575	CC	93 (93.00)	CT	1 (0.99)	TT	0 (0.00)	0.01	0.958651
chr21_35400771	AA	19 (22.27)	GA	54 (47.45)	GG	22 (25.27)	0.48	0.178679
chr21_43670900	AA	20 (18.32)	AG	43 (46.36)	GG	31 (29.32)	0.44	0.48269
chr21_49981840	CC	82 (80.52)	GC	10 (12.96)	GG	2 (0.52)	0.07	0.48269
chr22_15177905	AA	5 (4.30)	GA	30 (31.40)	GG	58 (57.30)	0.22	0.667675
chr22_27379570	TT	88 (88.07)	TA	5 (4.87)	AA	0 (0.07)	0.03	0.789932
chr22_33451883	AA	49 (48.27)	AG	36 (37.46)	GG	8 (7.27)	0.28	0.706586
chr22_42575267	GG	13 (12.80)	GT	43 (43.40)	TT	37 (36.80)	0.37	0.928612
chr22_43452738	CC	36 (35.55)	CT	43 (43.90)	TT	14 (13.55)	0.38	0.843637
chr22_48430418	CC	34 (30.78)	CT	39 (45.45)	TT	20 (16.78)	0.42	0.171348
chr22_6758970	CC	2 (4.69)	CT	38 (32.62)	TT	54 (56.69)	0.22	0.10958
chr23_14640451	AA	14 (10.45)	AT	34 (41.11)	TT	44 (40.45)	0.34	0.09719
chr23_19843835	CC	67 (68.21)	CT	27 (24.57)	TT	1 (2.21)	0.15	0.335867
chr23_33469971	CC	1 (1.05)	CT	18 (17.89)	TT	76 (76.05)	0.11	0.954279
chr24_19229425	AA	13 (14.72)	CA	48 (44.56)	CC	32 (33.72)	0.40	0.456463
chr24_2951565	CC	70 (68.82)	CT	20 (22.37)	TT	3 (1.82)	0.14	0.307728
chr24_41506662	CC	72 (72.41)	CT	21 (20.19)	TT	1 (1.41)	0.12	0.695886
chr24_45551575	AA	36 (31.35)	AG	36 (45.29)	GG	21 (16.35)	0.42	0.695886
chr24_47356206	CC	12 (13.79)	TC	48 (44.43)	TT	34 (35.79)	0.38	0.43534
chr24_838301	CC	12 (10.22)	TC	38 (41.55)	TT	44 (42.22)	0.33	0.407079
chr24_9893027	CC	34 (37.03)	TC	50 (43.94)	TT	10 (13.03)	0.37	0.180864
chr25_16680323	AA	18 (15.60)	AG	41 (45.79)	GG	36 (33.60)	0.41	0.307494
chr25_26796967	CC	80 (79.40)	CT	10 (11.21)	TT	1 (0.40)	0.07	0.303593

chr25_31572716	CC	62 (57.47)	CT	23 (32.06)	TT	9 (4.47)	0.22	0.303593
chr25_38556008	CC	23 (21.76)	TC	43 (45.48)	TT	25 (23.76)	0.49	0.603213
chr25_6476519	AA	38 (31.70)	GA	32 (44.61)	GG	22 (15.70)	0.41	0.603213
chr26_11865793	AA	15 (13.17)	GA	40 (43.66)	GG	38 (36.17)	0.38	0.419323
chr26_25527554	CC	2 (2.06)	CT	24 (23.87)	TT	69 (69.06)	0.15	0.958871
chr26_36767942	CC	92 (92.01)	CT	2 (1.98)	TT	0 (0.01)	0.01	0.91697
chr27_16796673	AA	11 (13.94)	GA	50 (44.13)	GG	32 (34.94)	0.39	0.199492
chr27_43327892	GG	72 (73.19)	GT	21 (18.63)	TT	0 (1.19)	0.11	0.219682
chr28_14684503	CC	1 (1.78)	TC	24 (22.44)	TT	70 (70.78)	0.14	0.498656
chr28_25324476	AA	56 (58.26)	AT	36 (31.49)	TT	2 (4.26)	0.21	0.164895
chr28_4239827	GG	75 (73.19)	GT	15 (18.63)	TT	3 (1.19)	0.11	0.060295
chr29_10012074	GG	91 (91.02)	GA	3 (2.95)	AA	0 (0.02)	0.02	0.87507
chr29_12133013	CC	51 (51.94)	CG	37 (35.12)	GG	5 (5.94)	0.25	0.606431
chr29_32494107	CC	68 (49.19)	CT	0 (37.62)	TT	26 (7.19)	0.28	0.606431
chr29_33757407	AA	7 (6.39)	AG	35 (36.23)	GG	52 (51.39)	0.26	0.742288
chr29_37306224	CC	25 (20.13)	CG	37 (46.74)	GG	32 (27.13)	0.46	0.742288
chr29_43972589	CC	74 (71.42)	TC	15 (20.16)	TT	4 (1.42)	0.12	0.742288
chr29_44457122	GG	91 (91.02)	GA	3 (2.95)	AA	0 (0.02)	0.02	0.87507
chr29_47978927	TT	80 (80.52)	TA	14 (12.96)	AA	0 (0.52)	0.07	0.43534
chr3_111833179	CC	28 (30.53)	CT	50 (44.93)	TT	14 (16.53)	0.42	0.279604
chr3_18320028	AA	67 (62.93)	GA	19 (27.15)	GG	7 (2.93)	0.18	0.279604
chr3_19819903	AA	84 (83.38)	AG	10 (11.24)	GG	1 (0.38)	0.06	0.281528
chr3_25191800	AA	8 (5.39)	GA	29 (34.23)	GG	57 (54.39)	0.24	0.138594
chr3_34248317	CC	64 (62.26)	CT	25 (28.48)	TT	5 (3.26)	0.19	0.235664
chr3_52560730	AA	1 (1.44)	AG	21 (20.12)	GG	70 (70.44)	0.13	0.676657
chr3_56826706	CC	24 (23.50)	CG	46 (47.00)	GG	24 (23.50)	0.50	0.836569
chr3_63703784	CC	82 (81.45)	TC	11 (12.10)	TT	1 (0.45)	0.07	0.377684
chr3_70933847	AA	11 (14.56)	CA	52 (44.87)	CC	31 (34.56)	0.39	0.12355
chr3_8244357	CC	10 (7.55)	GC	33 (37.90)	GG	50 (47.55)	0.28	0.212644
chr3_9561895	CC	72 (69.80)	TC	18 (22.40)	TT	4 (1.80)	0.14	0.05666
chr3_96987256	CC	41 (39.79)	CT	39 (41.43)	TT	12 (10.79)	0.34	0.573817

chr4_101405885	AA	70 (69.68)	GA	21 (21.64)	GG	2 (1.68)	0.13	0.775555
chr4_102029664	AA	44 (44.95)	GA	42 (40.11)	GG	8 (8.95)	0.31	0.647121
chr4_107062741	GG	11 (9.68)	GT	38 (40.65)	TT	44 (42.68)	0.32	0.530264
chr4_119159692	GG	86 (86.17)	CG	8 (7.66)	CC	0 (0.17)	0.04	0.666538
chr4_121362928	CC	49 (49.19)	TC	38 (37.62)	TT	7 (7.19)	0.28	0.92137
chr4_57902619	CC	94 (94.00)	TC	1 (0.99)	TT	0 (0.00)	0.01	0.958871
chr4_63072407	CC	44 (45.01)	CT	40 (37.98)	TT	7 (8.01)	0.30	0.611534
chr4_63120867	CC	57 (55.92)	CT	31 (33.16)	TT	6 (4.92)	0.23	0.526812
chr4_70547047	AA	70 (69.80)	GA	22 (22.40)	GG	2 (1.80)	0.14	0.861127
chr5_111347186	AA	71 (71.53)	GA	22 (20.94)	GG	1 (1.53)	0.13	0.62226
chr5_11447164	CC	23 (17.45)	CT	35 (46.10)	TT	36 (30.45)	0.43	0.62226
chr5_117022783	GG	73 (73.98)	AG	19 (17.04)	AA	0 (0.98)	0.10	0.269379
chr5_125355130	CC	48 (49.19)	CT	40 (37.62)	TT	6 (7.19)	0.28	0.539093
chr5_22044423	CC	2 (1.68)	TC	21 (21.64)	TT	70 (69.68)	0.13	0.775555
chr5_39634902	CC	11 (6.39)	TC	27 (36.23)	TT	56 (51.39)	0.26	0.775555
chr5_4660991	CC	46 (47.76)	CT	42 (38.49)	TT	6 (7.76)	0.29	0.376524
chr5_64770948	AA	12 (9.90)	GA	37 (41.21)	GG	45 (42.90)	0.32	0.322206
chr5_68051841	AA	16 (14.72)	AT	42 (44.56)	TT	35 (33.72)	0.40	0.579676
chr5_71562498	AA	42 (45.64)	GA	47 (39.72)	GG	5 (8.64)	0.30	0.075478
chr6_10506177	AA	26 (24.51)	AG	44 (46.98)	GG	24 (22.51)	0.49	0.538725
chr6_108831173	AA	60 (58.88)	GA	28 (30.24)	GG	5 (3.88)	0.20	0.475643
chr6_111027512	CC	1 (1.32)	TC	20 (19.37)	TT	71 (71.32)	0.12	0.754899
chr6_24363930	AA	7 (3.26)	GA	21 (28.48)	GG	66 (62.26)	0.19	0.754899
chr6_49453155	GG	92 (92.01)	GA	2 (1.98)	AA	0 (0.01)	0.01	0.91697
chr6_7179414	GG	88 (88.10)	GA	6 (5.81)	AA	0 (0.10)	0.03	0.749251
chr7_24546537	GG	85 (85.17)	AG	8 (7.66)	AA	0 (0.17)	0.04	0.664708
chr7_28660701	CC	17 (15.53)	CT	42 (44.95)	TT	34 (32.53)	0.41	0.527293
chr7_47310281	AA	54 (53.63)	GA	34 (34.74)	GG	6 (5.63)	0.24	0.835385
chr7_49827435	AA	54 (53.63)	GA	34 (34.74)	GG	6 (5.63)	monomorph	0.835385
chr7_53807884	AA	29 (23.75)	AG	36 (46.49)	GG	28 (22.75)	0.49	0.835385
chr7_57271796	AA	92 (92.00)	GA	1 (0.99)	GG	0 (0.00)	0.01	0.958427

chr7_99428251	GG	8 (6.99)	GT	35 (37.02)	TT	50 (48.99)	0.27	0.599407
chr8_14993490	AA	49 (51.94)	AG	41 (35.12)	GG	3 (5.94)	0.25	0.106652
chr8_63200053	CC	4 (4.30)	CT	32 (31.40)	TT	57 (57.30)	0.22	0.853271
chr8_66401358	CC	7 (11.94)	CT	53 (43.12)	TT	34 (38.94)	0.36	0.853271
chr8_87985395	GG	92 (92.00)	GA	1 (0.99)	AA	0 (0.00)	0.01	0.958427
chr8_93194158	CC	10 (11.81)	CT	47 (43.37)	TT	38 (39.81)	0.35	0.415133
chr9_38921793	AA	32 (35.32)	GA	50 (43.37)	GG	10 (13.32)	0.38	0.142541
chr9_40147943	AA	26 (16.96)	GA	27 (45.08)	GG	39 (29.96)	0.43	0.142541
chr9_6302504	CC	65 (62.11)	CT	22 (27.78)	TT	6 (3.11)	0.18	0.142541
chr9_8662828	AA	17 (16.18)	GA	44 (45.64)	GG	33 (32.18)	0.41	0.727811
chr9_87183394	TT	83 (83.32)	TC	11 (10.36)	CC	0 (0.32)	0.06	0.546817
chrX_64976718	CC	2 (0.04)	CG	0 (3.91)	GG	91 (89.04)	0.02	0.546817
chrX_87268108	CC	1 (0.87)	TC	16 (16.26)	TT	76 (75.87)	0.10	0.87834
