# Supplementary material for: Whole genome sequencing of a single Bos taurus animal for single nucleotide polymorphism discovery
Source: Genome Biol. 2009 Aug 6;10(8):R82. doi: 10.1186/gb-2009-10-8-r82 (PMC2745763; doi:10.1186/gb-2009-10-8-r82)
Supplement: Additional data file 4 — Calls by MAQ were checked by MALDI-TOF spectroscopy and capillary sequencing. [file gb-2009-10-8-r82-S4.pdf]

Addtional file 4. False-positive SNP calls in 75 coding SNPs with high read depth (>=16). Calls by MAQ were checked by MALDI-TOF mass spectroscopy and capillary sequencing.

Position	MAQ	MALDI-TOF	MALDI-TOF genotype frequency	Capillary sequencing	Reference allele	Sequence depth	Frequency of reads in MAQ
chr3_16650156	AG	AG	96 of 96	A	A	19	G=0.26
chr4_117247234	CT	TT	96 of 96	PCR failed (double band)	T	19	C=0.58
chr4_117247581	AG	AA	96 of 96	Seq failed	A	20	G=0.35
chr6_118775387	AG	AA	95 of 95	A	A	18	G=0.39
chr6_118775516	AG	AA	96 of 96	A	A	16	G=0.38
chr10_62623710	AG	AG	96 of 96	A	A	20	G=0.45
chr11_14722157	AG	AA	96 of 96	A	A	18	G=0.44
chr12_56595781	AT	AA	96 of 96	A	A	19	T=0.26
chr12_56596185	AG	AA	96 of 96	A	A	18	G=0.56
chr13_16920248	CT	CT	95 of 95	PCR failed	T	19	C=0.68
chr13_27573781	AG	AG	96 of 96	AG (12 of 12 animals)	G	18	A=0.39
chr13_27573821	CG	CC	96 of 96	C	C	17	G=0.35
chr13_38556365	AG	AA	96 of 96	A	A	19	G=0.32
chr13_64445178	CT	CT	95 of 95	C	C	19	T=0.63
chr17_74621895	AG	AG	95 of 95	A	A	20	G=0.45
chr19_26939072	AC	CC	96 of 96	C	C	18	A=0.44
chr23_27109842	AG	AG	95 of 95	G (seq failed in 7 of 12 animals)	G	20	A=0.55
chr25_29302062	AT	AA	96 of 96	A	A	17	T=0.29
